# Eddy Current Position Measurement in Harsh Environments: A Temperature Compensation and Calibration Approach

**DOI:** 10.3390/s24051483

**Published:** 2024-02-24

**Authors:** Gabriel Gruber, Bernhard Schweighofer, Matthias Berger, Thomas Leitner, Gerald Kloesch, Hannes Wegleiter

**Affiliations:** 1 Christian Doppler Laboratory for Measurement Systems for Harsh Operating Conditions, Institute of Electrical Measurement and Sensor Systems, Graz University of Technology, 8010 Graz, Austria; bernhard.schweighofer@tugraz.at (B.S.); wegleiter@tugraz.at (H.W.); 2 Voestalpine Stahl Donawitz GmbH, 8070 Leoben, Austria; matthias.berger@voestalpine.com (M.B.); t.leitner@voestalpine.com (T.L.); gerald.kloesch@voestalpine.com (G.K.)

**Keywords:** eddy current displacement sensor, position measurement, harsh environment, large displacements, high temperature variations, vaporization effects, sensor design

## Abstract

Eddy current displacement sensors (ECDSs) are widely used for the noncontact position measurement of small displacements (lift-offs). Challenges arise with larger displacements as the sensitivity of the ECDSs decreases. This leads to a more pronounced impact of temperature variations on the inductance and, consequently, an increased position error. Design solutions often rely on multiple coils, suitable coil carrier materials, and compensation measures to address the challenges. This study presents a single-coil ECDS for large displacement ranges in environments with high temperatures and temperature variations. The analysis is based on a sensor model derived from an equivalent circuit model (ECM). We propose design measures for both the sensing coil and the target, focusing on material selection to handle the impact of temperature variations. A key part of improving performance under varying temperatures includes model-based temperature compensation for the inductance of the sensing coil. We introduce a method to calibrate the sensor for large displacements, using a modified coupling coefficient based on field simulation data. Our analysis shows that this single-coil ECDS design maintains a position error of less than 0.2% full-scale for a temperature variation of 100 K for the sensing coil and 110 K for the target.

## 1. Introduction

**Figure 1:**
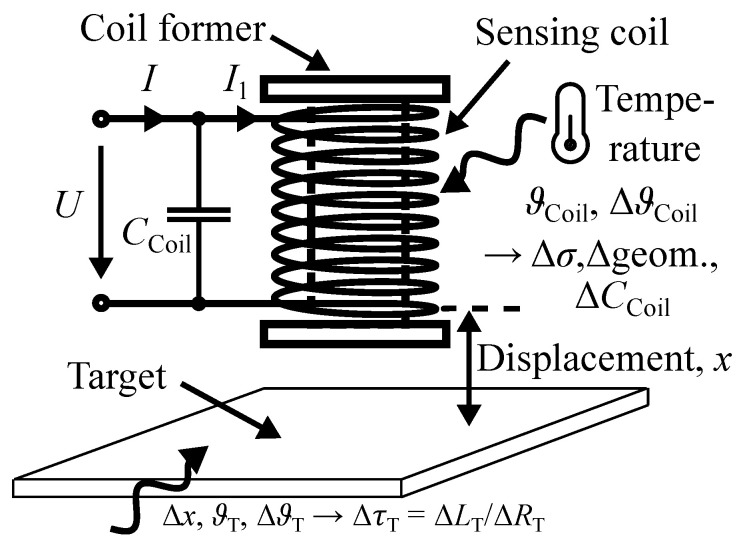
Sketch of an ECDS, detailing the sensing coil, target, and effects in harsh environments.

Eddy current displacement sensors (ECDSs) are established position sensors [1,2] and are robust against harsh environments, such as dirt, dust, and moisture [3,4,5]. Their contactless and compact design makes them widely used [6,7,8,9]. Figure 1 depicts a typical ECDS, comprising a sensing coil and a target. The displacement *x* between the sensing coil and the target is determined from changes in the impedance Z=U/I, particularly the change in inductance L=imag{Z}/ω [8,9,10,11,12].

For small displacement ranges, i.e., displacements less than the coil radius (x<r), within low temperature ranges (ϑ<50 °C), ECDSs show remarkable performance [8,13].

Challenges arise for large displacement ranges (x>r), where the ECDS operates in a range with low sensitivity. Using multiple coils is an approach to increase sensitivity while also improving linearity and the thermal drift coefficient [14,15]. A common approach, using a single coil, is scaling up the sensor dimensions so that x<r, allowing the sensor to operate in a measurement range with higher sensitivity. However, larger sensor dimensions increase cross-sensitivities with respect to environmental influences. For example, temperature or humidity changes can affect different parts of large sensors unevenly, while smaller sensors tend to be affected uniformly.

Temperature variations have a significant impact on the inductance *L* of an ECDS as they change the electrical conductivity (Δσ), the thermal expansion (Δgeom.), and the distributed parasitic capacitance $C_{\mathrm{Coil}}$ via humidity vaporization. They also affect the material properties of the target ($\Delta \tau_{T}$). These temperature-related effects are highlighted in Figure 1. The temperature stability (TS) is a potential metric to evaluate an ECDS.

Given the effect of temperature variations on the inductance *L*, accurate determination of the position *x* is required. To determine the position *x*, the sensor model in Equation (2) is transformed to k(x). For this, the inductance $L_{Coil}=imag\left\{U/I_{1}\right\}/\omega$ of the coil in air (in the absence of the target) and the fraction term $f_{T}$ must be known precisely. However, temperature variations significantly change both the inductance of the coil $L_{Coil}$ and the fraction term $f_{T}$ [13,16,17]. Particularly, this is so in the presence of ferromagnetic core materials, as in magnetic reluctance sensors, due to changes in the relative permeability. Their impact can be reduced by the sensor design, e.g., a mechanical design solution for the coil former or the use of a reference coil [8,18]. However, it is not possible to completely eliminate temperature effects by the sensor design alone. Therefore, temperature models that include terms such as $\left(1+\alpha \Delta \vartheta +\beta \Delta \vartheta^{2}\right)$ for the sensing coil can potentially be used for compensation. α and β are temperature coefficients and ϑ is the temperature of the sensing coil.

A precise determination of the position *x* is inherently linked to the TS of the sensor. Most academic research studies on ECDSs achieve a high TS by a trade-off between large displacement ranges and high temperature variations [8,14,15,18]. A detailed discussion of different research studies on ECDSs is provided in Appendix A. Refs. [19,20,21] also found that the TS is displacement-dependent, as the sensitivity changes.

Industrial manufacturers offer customized ECDSs, characterized by an ashtray-like design, for these specific applications. Table 1 lists the specification and TS of customized ECDSs from industry reports. The results of this work are also listed for comparison. Nevertheless, achieving a high TS for ECDSs remains a challenge in applications with both large displacement ranges and high temperature variations.

Our research presents an ECDS demonstrator for large displacement ranges and environments characterized by high temperatures and temperature variations. We use a single coil design and achieve a TS that matches or exceeds that of both commercially available ECDSs and published academic research studies on ECDS. We also differ from those comparative studies by providing separate TS for the sensing coil and the target, as shown in Table 1.

**Figure 2:**
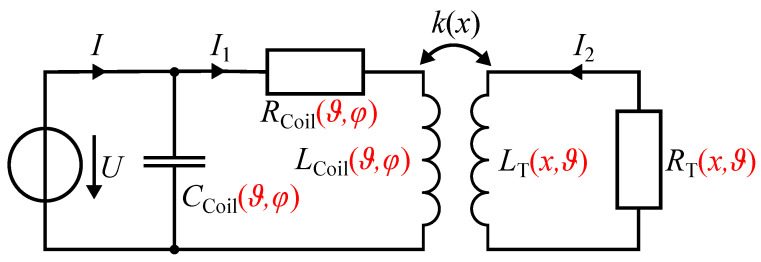
Harsh environment ECM (dependencies marked in red) of an ECDS.

In this study, we analyze the properties of an ECDS for applications with large displacements (x>100 mm), high temperatures (ϑ>100 °C), and high temperature variations (Δϑ>100 K). The sensor model in (2) and the equivalent circuit model (ECM) shown in Figure 2 form the basis for our analysis. We separately highlight the impact of high temperatures and temperature variations in the sensing coil and the target on the inductance *L* through simulation studies and comparative measurements. To reduce their impact, we propose design measures for the sensing coil and the target. We found that vaporization effects affect the parasitic capacitance $C_{\mathrm{Coil}}$ and thus the impedance *Z*. To determine the impact on the coil resonance frequency, we perform an impedance spectroscopy measurement during heating. The analysis is carried out at two different frequencies, i.e., at 500 Hz and 15 kHz. We present a calibration approach for large displacements using a simulation-based model description. Finally, we address the position error $e_{\mathrm{pos}.}$ due to the temperature variation and the TS of both the sensing coil and the target. By taking design measures, the TS of ECDSs with a single coil configuration can be improved in harsh environments.

The main contributions and novelty of this work are as follows:

- A holistic analysis for all the system components of an ECDS with respect to the sensor model in (2) and the ECM in Figure 2, which comprises the following:
  – The displacement and temperature dependence of the target.
  – A temperature characterization of the sensing coil.
  – An investigation of the influence of parasitic effects based on the coil manufacturing technique.
- Countermeasures to reduce the impact of temperature variations on an ECDS.
- The TS in ppm FS/K for the sensing coil and the target of an ECDS.

## 2. Analysis of an Eddy Current Displacement Sensor

In this section, we present an ECM for harsh environments and derive the sensor model for the inductance $L_{ECDS}$. We show two prototypes for the coil carrier made from different materials, namely, gypsum and fired clay, both suitable for high-temperature applications. In addition, we detail the lab setup and a field simulation with a focus on the flux lines. We also present a comparison between the measured and simulated inductance *L* for two different target materials, steel and copper, at two different frequencies. The sensitivity of the ECDS and the material properties of the target in relation to $\tau_{T}$ are discussed.

### 2.1. Equivalent Circuit Model of the ECDS in Harsh Environments

For an electrical description of an ECDS, the ECM shown in Figure 2, without the red-marked dependencies, is commonly used [8,22,23]. The primary side represents the sensing coil ($R_{\mathrm{Coil}}$, $L_{\mathrm{Coil}}$), and the secondary side represents the target ($R_{T}$, $L_{T}$). The parallel capacitor $C_{\mathrm{Coil}}$ represents the distributed interwinding capacitance in interaction with the dielectric properties of the coil former. When using nonhygroscopic materials, the parasitic capacitance $C_{\mathrm{Coil}}$ is mostly neglected [8,24].

In harsh environments, the temperature and humidity dependence of the sensing coil, as well as the temperature and displacement dependence of the target, must be considered. These dependencies are marked in red in the ECM shown in Figure 2. Note that the frequency dependence is not mentioned separately. The EMC assumes uniform and gradual effects over the entire sensor. Local effects or extreme gradients that affect only parts of the coil or target can lead to discrepancies in model predictions.

The impedance Z=U/I is measured and depends on the displacement, the geometry of the sensing coil and the target, the temperature, the humidity, and the measurement frequency. Based on the ECM in Figure 2, the impedance of the sensor, $Z_{\mathrm{ECDS}}$, neglecting the capacitance $C_{\mathrm{Coil}}$, can be derived as follows

(1) $$\begin{array}{c} Z_{\mathrm{ECDS}}=\frac{U}{I_{1}}=\underset{1}{\underset{︸}{R_{\mathrm{coil}}+j\omega L_{\mathrm{coil}}}}+\underset{2}{\underset{︸}{\left(R_{T}-j\omega L_{T}\right)\;\cdot \;k{\left(x\right)}^{2}\;\cdot \;\frac{\omega^{2}L_{\mathrm{coil}}L_{T}}{R_{T}^{2}+{\left(\omega L_{T}\right)}^{2}}}} \end{array}$$

and was previously determined in [9,22]. The first term in Equation (1) describes the influence of the sensing coil, and the second term in Equation (1) describes the joint influence of the target and the sensing coil on the impedance $Z_{\mathrm{ECDS}}$. k(x) is the coupling coefficient between the sensing coil and the target. The coupling coefficient k(x) is non-linear and exhibits a significant decrease with displacement. Nevertheless, k(x) can later potentially be used for calibration.

From the impedance $Z_{\mathrm{ECDS}}$, we calculate the inductance $L_{\mathrm{ECDS}}$ and substitute $L_{T}/R_{T}$ with the time constant $\tau_{T}$ [9,11]. The inductance of the sensor $L_{\mathrm{ECDS}}$ can be described by the sensor model

(2) $$\begin{array}{c} L_{\mathrm{ECDS}}=L_{\mathrm{Coil}}\;\cdot \;\left(1-k{\left(x\right)}^{2}\;\cdot \;\underset{f_{T}}{\underset{︸}{\frac{{\left(\omega \tau_{T}\right)}^{2}}{1+{\left(\omega \tau_{T}\right)}^{2}}}}\right), \end{array}$$

where $L_{\mathrm{Coil}}$ describes the inductance of the air coil (in the absence of the target) that takes temperature drifts into account. The time constant $\tau_{T}$ represents the electrical and magnetic properties of the target. This model description is found in [9,11]. k(x) can be seen as the coupling coefficient of an ideal ECDS, i.e., an ECDS that measures the distance to a target of infinite conductivity ($\tau_{T}\to \infty$). The product term $k{\left(x\right)}^{2}f_{T}$ describes the properties of a real ECDS.

The corresponding expression for the resistance $R_{\mathrm{ECDS}}$ is given by

(3) $$\begin{array}{cc} R_{\mathrm{ECDS}} & =R_{\mathrm{coil}}\;\cdot \;\left(1+k{\left(x\right)}^{2}\;\cdot \;\underset{f_{T_{R}}}{\underset{︸}{\frac{\omega^{2}\tau_{T}\tau_{\mathrm{coil}}}{1+{\left(\omega \tau_{T}\right)}^{2}}}}\right) \end{array}$$

where $\tau_{\mathrm{coil}}=L_{\mathrm{coil}}/R_{\mathrm{coil}}$. The resistance $R_{\mathrm{ECDS}}$ also depends on the displacement *x* and can be used for the position evaluation, as shown in [8]. However, the resistance is impacted severely by temperature, due to the temperature coefficient of 3900 ppm/°C for copper wire coil, which needs to be compensated [8,18].

By choosing the product term $\omega \tau_{T}\gg 1$, as recommended in [8,9], the inductance $L_{\mathrm{ECDS}}$ becomes more insensitive with respect to the properties of the target. For the resistance $R_{\mathrm{ECDS}}$, a dependence of the target and the sensing coil remains in the fraction term $f_{T_{R}}$ in Equation (3). Therefore, we use the inductance $L_{\mathrm{ECDS}}$ to determine the position *x* and to assess the effects of temperature variations. With a measurement frequency of f=15 kHz and the use of a copper target ($\tau_{T}$ ∼ 100 μs as shown in Section 2.2.2), this condition can be met because $\omega \tau_{T}$ ∼ 9.5.

### 2.2. Sensing Coil and Lab Setup

Temperature-dependent effects on the inductance of the coil $L_{Coil}$ should be reduced by the sensing coil design, i.e., the coil former material and shape. The relevant coil former material is robust to harsh environments and hence can withstand high temperatures and has minimal thermal expansion, low humidity absorption, and minimal eddy current effects, i.e., nonmagnetic materials with low electrical conductivity.

Technical ceramics, such as alumina and silicon carbide, or glass ceramics, such as Macor^®^ or Mica^®^, are viable options for high-temperature applications. However, these materials require significant manufacturing efforts and are cost-intensive, making them impractical for extensive prototyping and parameter studies. In this study, we address two coil prototypes out of several prototypes manufactured in-house. The coil formers of these prototypes are made of different materials, namely, gypsum and fired clay. Gypsum is widely available, easy to manufacture, and low cost; possesses a low electrical conductivity; and is nonmagnetic. Fired clay shares properties similar to technical ceramics and has the additional advantage over gypsum of withstanding temperatures of up to 1200 °C and exhibiting minimal thermal expansion [25]. The ease of manufacture is a major advantage over technical ceramics, particularly in prototyping, when promising simulation results can be verified rapidly with a prototype in the laboratory. The behavior of gypsum and fired clay under temperature variations broadly reflects that of technical ceramics. Having identified the effects of temperature variation on the inductance, we can implement countermeasures that also apply to technical ceramics. For the final application, we proceed with technical ceramics and utilize their enhanced properties.

Using a finite element analysis in COMSOL Multiphysics^®^, we determine the coil dimensions, winding cross-section, number of turns, and number of layers to achieve a high sensitivity dL/dx. For our specific application, the analysis results in the following coil specifications: The inner diameter of the sensing coil’s cross-sections is approximately 110 mm and the winding cross-section has a width of 20 mm and a height of 3 mm. The outer diameter of the sensing coil’s cross-sections is about 150 mm. The sensing coil has 500 turns with a wire diameter of $d_{\mathrm{Wire}}$ = 0.3 mm.

**Figure 3:**
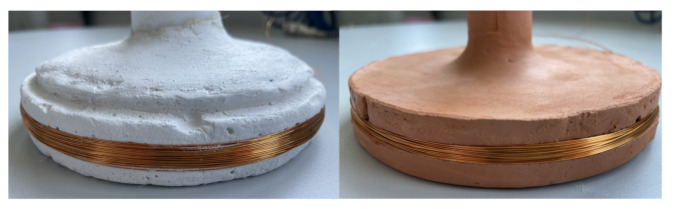
Photo of the two sensing coil prototypes made of gypsum (**left**) and fired clay (**right**).

The two sensing coil prototypes are shown in Figure 3 and include a round gypsum (left side) and a round fired clay-based (right side) former. A groove has been made in the sensing coil former to hold the copper winding. The coil is compactly wound, but the turns are randomly distributed in the groove from a certain number of layers. They have an inductance *L* of approximately 57 mH at a frequency of 15 kHz.

#### 2.2.1. Lab Setup for Displacement Experiments

**Figure 4:**
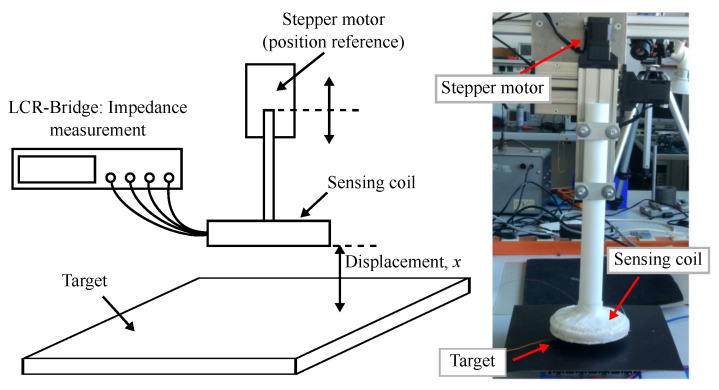
Sketch and photo of the lab setup for the displacement experiments.

Figure 4 shows a sketch (left side) and a photo (right side) of the lab setup for the displacement experiments. The sensing coil is mounted on a sliding table, a stepper motor-based positioning system. The sliding table serves as a position reference with an accuracy in the low tens of μm. The accuracy of the sliding table is verified using a Keyence IL-065 laser position sensor. With the lab setup we investigate the response of the ECDS over a displacement range of 20 mm to 100 mm.

The impedance *Z* is measured with an LCR-bridge (Hameg HM8118) at frequencies of 500 Hz and 15 kHz. From the impedance *Z*, we calculate the inductance *L*. Note that the frequencies are set one order of magnitude below the resonance frequencies of the coils. This ensures that the coils operate within the inductive region of the impedance characteristic. We use the highest measurement voltage of 1.5 V and the slowest measurement speed to ensure maximum accuracy of the measurement.

#### 2.2.2. Comparison of the Inductance *L* between Measurement and Simulation

**Figure 5:**
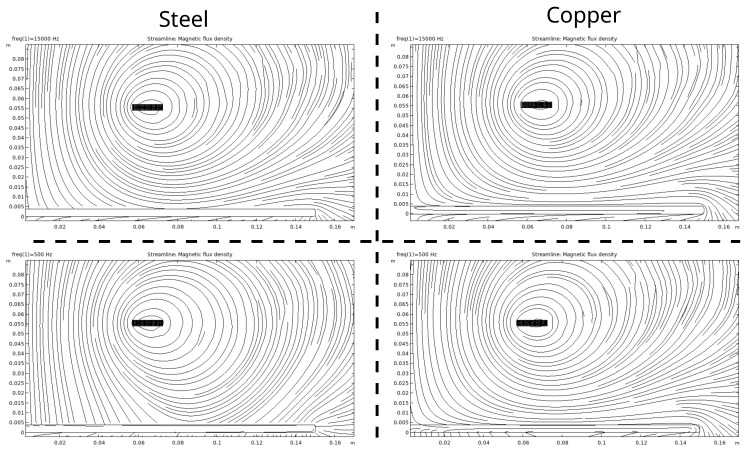
Flux lines of the FEM simulation for steel (**left half**) and copper (**right half**) targets at frequencies of 500 Hz (**lower half**) and 15 kHz (**upper half**).

**Figure 6:**
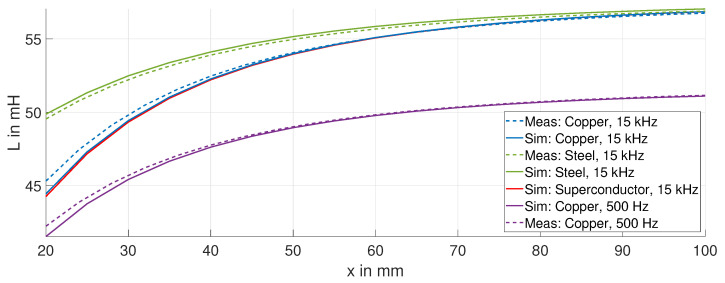
Simulation and measurement of the inductance *L* for different target materials, e.g., steel, copper, and a superconductor (simulation only).

Figure 5 shows the 2D axisymmetric field simulation model consisting of the sensing coil and the target. The sensing coil is modeled to closely match the actual dimensions of the fired clay prototype. The sensing coil is designed with single turns instead of a homogenized multiturn model with uniform current distribution, thus considering the skin effect. A limitation of this model is its compact winding, which maintains a constant number of turns per layer. This is unlike the coil on the fired clay former, which has distributed windings beyond a certain layer. Nevertheless, the simulation results closely match the measurements, as shown in Figure 6, indicating minimal deviation.

The flux lines for the steel (left half) and copper (right half) targets at frequencies of 500 Hz (lower half) and 15 kHz (upper half) are also shown in Figure 5. For the steel target, the flux lines converge toward the target, especially in the inner region of the coil. This is due to steel’s magnetic properties, which attract and channel magnetic flux lines. In contrast, for the copper target, the flux lines align parallel to the surface, indicating limited penetration into the material. As the frequency decreases, the skin depth increases, allowing for deeper magnetic field penetration.

Figure 6 shows the measured (dashed curves) and simulated (solid curves) inductance *L* of the ECDS on the fired clay-based coil former. The measurements are performed at room temperature. The inductance *L* of the gypsum former is close to that of the fired clay-based former and is therefore not included. The results of the displacement experiment for each target material and both frequencies are discussed below.

#### Discussion of the Displacement Experiment

As the sensing coil moves away from the target, the inductance *L* increases. For displacements greater than the radius of the sensing coil, the change in inductance ΔL decreases. The inductance *L* approaches that of the air coil $L_{Coil}$, for the specific frequency, as the displacement is further increased. There is a minor deviation between the simulation (solid curves) and measurement data (dashed curves). This is due to the imperfect matching geometry of the sensing coils and the distribution of the winding. However, the results are sufficiently similar to use the simulation model for scaling purposes.

We observe a reduced sensitivity using the steel target (green curve) compared to the copper target (blue curve), due to its lower conductivity $\sigma_{T}$ and the additional permeability $\mu_{T}$. For steel, $\tau_{T}$ is approximately 3 μs, and significantly smaller than for copper ($\tau_{T}$ ∼ 100 μs), and therefore the change in inductance ΔL is smaller. The ideal target material is a superconductor, as $\tau_{T}\to \infty$. The trend of the inductance *L* for copper is very close to the superconductor (red curve), highlighting the exceptional properties of copper as a target material.

At lower frequencies, the change in inductance for the copper target is smaller. This effect is illustrated by comparing the purple curve at 500 Hz with the blue curve at 15 kHz.

**Figure 7:**
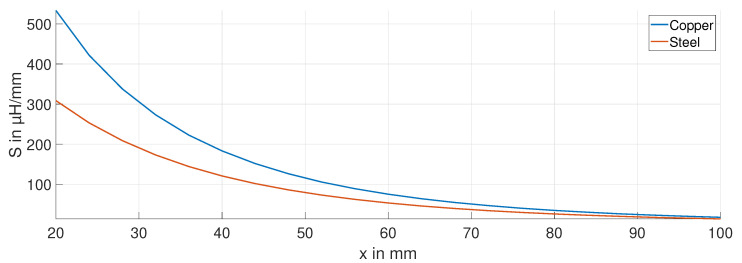
Sensitivity *S* for the steel and copper targets.

The sensitivity S=dL/dx of the ECDS is calculated from the measurement data for the copper (blue curve) and steel (red curve) targets at a frequency of 15 kHz and is shown in Figure 7. With increasing displacement, the sensitivity decreases. By using a copper target compared to a steel target, the sensitivity is approximately 70% higher over the entire displacement range.

## 3. Effects of Changes in the Material Properties of the Target on the Inductance *L*

In this section, we determine the effects of the changes in the material properties of the target on the inductance *L* and thus the position error due to temperature and displacement variations. Therefore, we perform simulations and comparative measurements. Furthermore, we use the sensitivity *S* to estimate the influence of $\tau_{T}$ on the position error.

### 3.1. Temperature Variations—A Simulation Study

**Figure 8:**
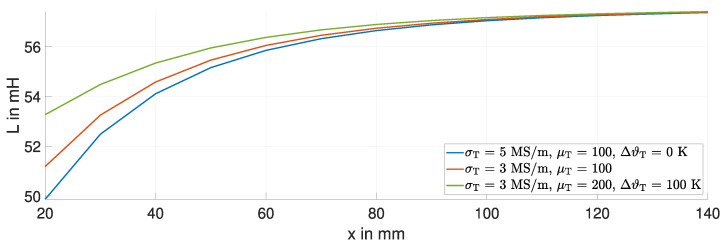
Simulated inductance *L* for a temperature variation of 100 K of the steel target.

When considering a temperature variation of approximately 100 K for the steel target, two effects are significant. First, the electrical conductivity σ of the used steel plate decreases with increasing temperature, from 5 MS/m to 3 MS/m. Second, the magnetic permeability μ increases as the temperature increases, from 100 to 200. These temperature-dependent changes in the material properties are based on findings by [26]. Figure 8 shows the simulated inductance *L* over the displacement *x* for this temperature variation at a frequency of 15 kHz.

As $\sigma_{T}$ decreases, the change in inductance ΔL decreases, e.g., the inductance *L* changes from the blue curve to the red curve. Note that the permeability of the target is held constant. The additional increase in $\mu_{T}$ decreases the change in inductance ΔL further, e.g., the inductance *L* changes from the red curve to the green curve. Especially below 60 mm, a temperature variation in the target significantly affects the inductance *L*.

**Figure 9:**
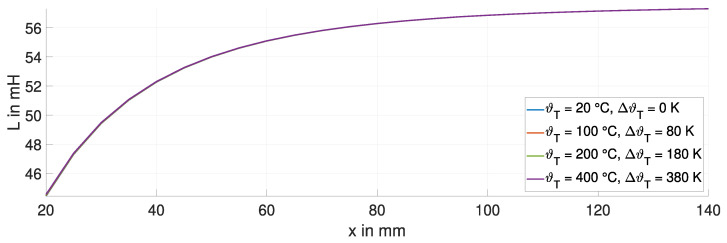
Simulated inductance *L* for a temperature variation of 380 K of the copper target.

For copper, the electrical conductivity σ is well known over a wide temperature range compared to steel [27]. A simulation with temperature variations of up to 380 K and more is possible. Copper is nonmagnetic, so only the electrical conductivity σ is varied. Figure 9 shows the corresponding inductances *L* at a frequency of 15 kHz. Despite the greater temperature variation in the copper target, the change in inductance ΔL is significantly less compared to the change in inductance due to temperature variations in the steel plate.

We use the field simulation to determine the order of magnitude of $\tau_{T}$ for the copper and steel targets. For the copper target, we show how temperature variations affect the material properties of the target, impacting the inductance *L*. Therefore, the sensor model in Equation (2) is transformed to $\tau_{T}$, where $L_{0}$ represents the inductance of the air coil only, and k(x) is evaluated from a simulation with a superconducting target.

**Figure 10:**
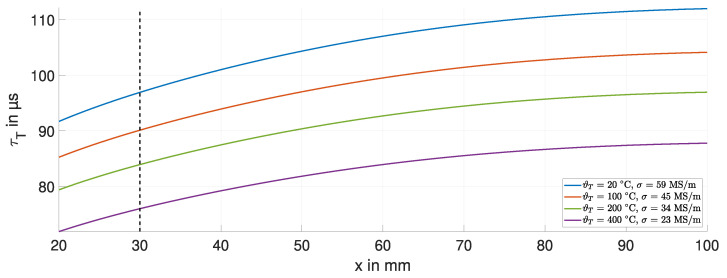
Temperature and displacement dependence of $\tau_{T}$ of the copper target.

Figure 10 shows $\tau_{T}$ for the copper target for a temperature variation of 380 K. At room temperature, $\tau_{\mathrm{Cu}}$ ∼ 100 μs. At higher temperatures, $\tau_{T}$ decreases due to the decrease in electrical conductivity σ. The simulation shows that the material properties of the target, represented by $\tau_{T}$, are also displacement-dependent.

At a displacement of x=30 mm, the change in $\tau_{T}$ is approximately 20 μs for the temperature variation of 380 K. This corresponds to a 20% decrease. This decrease in $\tau_{T}$ results in a change in the inductance ΔL of 50 μH based on Equation (2).

The simulation for the steel target shows that $\tau_{T}$ is approximately 3 μs at room temperature. Taking Equation (2), a comparative change in $\tau_{T}$ of about 20% results in a change in the inductance ΔL of 200 μH. However, we expect the change in $\tau_{T}$ to be higher even for temperature variations smaller than 380 K, which makes this estimation the best case. The resulting change in inductance ΔL is four times greater than that of the copper target. These simulation results confirm the interpretations of the product term $\omega \tau_{T}$ in Section 2.1. The larger this product term, the less effect temperature variations in the target have on the inductance *L*. Furthermore, if the temperature is known, $\tau_{T}$ can potentially be used to estimate the material properties of the target.

### 3.2. Temperature Variations—Experiments with a Steel and Copper Target

**Figure 11:**
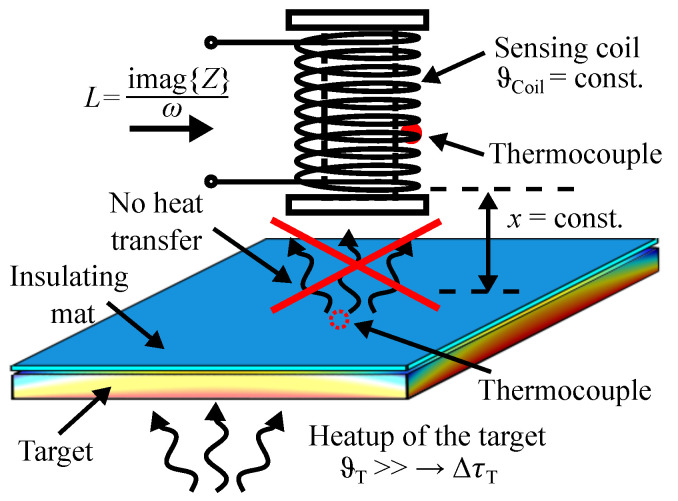
Sketch of the lab setup for temperature variations in the target $\Delta \vartheta_{T}$.

In the following experiments, we analyze the impact of temperature variations in the target $\Delta \vartheta_{T}$ on the inductance *L* at frequencies of 500 Hz and 15 kHz. Figure 11 shows a sketch of the lab setup. We attach heating resistors to the bottom of the target for controlled heating. To minimize heat transfer to the sensing coil, an insulating mat is placed on top of the target. With this setup, the target can be heated up to 150 °C in a controlled lab environment. To monitor the temperature of both the target and the sensing coil, we use thermocouples and a thermal camera. A thermal steady-state of both the target and the sensing coil is maintained before displacing the sensing coil from 20 mm to 100 mm.

**Figure 12:**
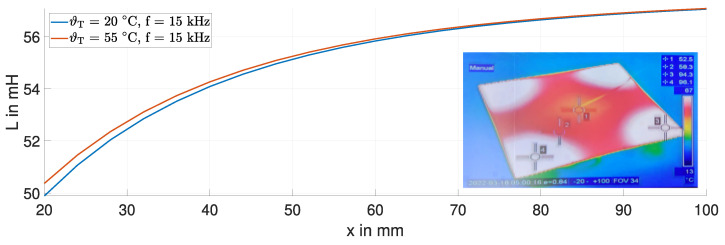
Measured inductance *L* and thermal image of the steel plate at 15 kHz.

Figure 12 shows the measured inductance *L* at room temperature and at a temperature of 55 °C for the steel target at a frequency of 15 kHz. The thermal image, the inlet in Figure 12, shows the thermal steady-state of the steel target. Due to the low thermal conductivity of steel (45 W K/m), a temperature gradient remains from the center of the target to the heating spots in the corners. The area directly beneath the sensing coil has the greatest influence on the change in inductance. Here, the average temperature of the steel target is about 55 °C. The target experienced minimal bending during heating. Hence, the significant change in the inductance *L*, particularly for small displacements (x<50 mm), can be attributed to changes in the electrical conductivity and magnetic permeability of the steel target.

**Figure 13:**
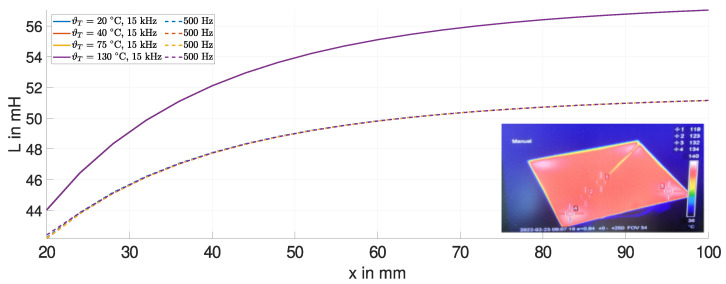
Measured inductance *L* and thermal image of the copper plate at 500 Hz and 15 kHz.

Figure 13 shows the measured inductance *L* for the copper target at room temperature and at a temperature of 130 °C for frequencies of 500 Hz (dashed curves) and 15 kHz (solid curves). The thermal image shows a uniform temperature distribution of the copper target due to its higher thermal conductivity (400 W K/m).

The highest temperature of the target caused a slight downward bending of its outer edges, affecting the proximity of the coil to the target. Consequently, the inductance *L* is marginally lower, as we did not adjust the distance between the sensing coil and the target. This change in the inductance *L* is less pronounced compared to the steel target at both frequencies. Nevertheless, this minor temperature-induced effect can lead to a significant position error $e_{\mathrm{pos}.}$, which needs to be investigated. According to the sensor model in Equation (2), the temperature-dependent change in the inductance *L* at x=20 mm and a frequency of 500 Hz is five times higher than at 15 kHz. This discrepancy is due to the significant decrease in $f_{T}$ by approximately 90% with a decreasing frequency. Thus, the influence of temperature-dependent changes in the material properties of the target on the inductance *L* is amplified. However, the effects of bending and the change in $\tau_{T}$ on the inductance *L* are counteractive, resulting in a minor change in inductance *L* at 500 Hz.

### 3.3. Target: Temperature-Induced Inductance Variations—Determination of the Position Error

In this section, we evaluate the effects of temperature variations in the target on the inductance *L* and determine the position error $e_{\mathrm{pos}.}$. The evaluation is based on the measurement data of the inductance *L*. Therefore, we calculate the change in the inductance $\Delta L=L\left(\vartheta_{T}\right)-L_{0,x=20\mathrm{mm}}$, where $L_{0,x=20\mathrm{mm}}$ is the inductance at room temperature at a displacement of x=20 mm. To translate the change in the inductance ΔL into a quantifiable position $x_{T}=\Delta L/S$, we use the sensitivity *S*. With this, we calculate the position error $e_{\mathrm{pos}.}=x_{T}-x_{\mathrm{Ref}.}$, where $x_{\mathrm{Ref}.}$ is the reference position of the stepper motor.

**Figure 14:**
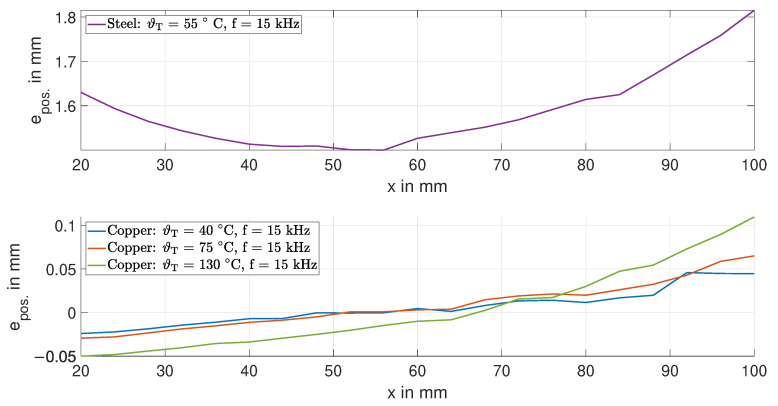
Position error $e_{\mathrm{pos}.}$ due to temperature variations in the steel and copper targets at 15 kHz.

Figure 14 shows the corresponding position error $e_{\mathrm{pos}.}$ for the steel (upper subplot) and copper (lower subplot) targets at a frequency of 15 kHz. The position error $e_{\mathrm{pos}.}$ for the copper target is less than 0.1 mm. For the steel target, the position error is 10 times that of the copper target, although the temperature change of the steel target is only 30%. Note that, for the copper target, the position error $e_{\mathrm{pos}.}$ for displacements smaller than 50 mm is negative. This is due to the effects of bending.

**Figure 15:**
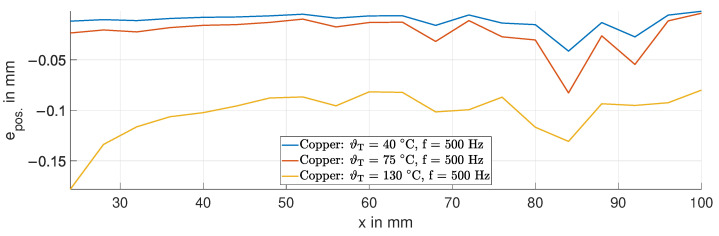
Position error $e_{\mathrm{pos}.}$ due to temperature variations in the copper target at 500 Hz.

Figure 15 shows the position error $e_{\mathrm{pos}.}$ for the copper target at a frequency of 500 Hz. The position error is in the range of 0.15 mm. The effect of the bending of the target is also evident in the position error, such as at 15 kHz.

## 4. Characterization of the Sensing Coil and a Compensation Approach

**Figure 16:**
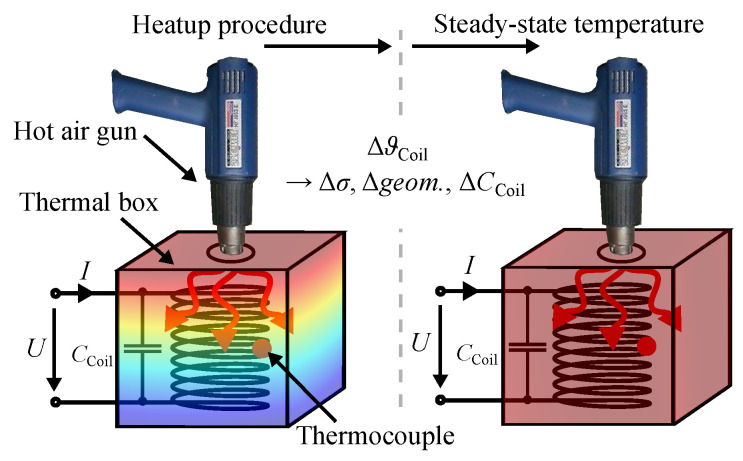
Sketch of the lab setup for heating the sensing coil with a hot air gun.

In this section, we analyze the temperature dependence of the inductance $L_{\mathrm{Coil}}=imag\left\{U/I\right\}/\omega$ (according to Figure 16) of the sensing coil in air, in the absence of the target. This is performed for gypsum and fired clay prototypes at frequencies of 500 Hz and 15 kHz. We introduce a potential temperature compensation model and highlight the effect of humidity vaporization on the inductance $L_{\mathrm{Coil}}$ by using impedance spectroscopy measurements. Finally, we show the effect of the temperature variations on the inductance *L* of the ECDS (Figure 1) and determine the resulting position error $e_{\mathrm{pos}.}$.

### 4.1. Model Approach for the Sensing Coil

Temperature variations in the sensing coil affect the electrical conductivity (Δσ), the thermal expansion (Δgeom.), and the parasitic capacitance $C_{\mathrm{Coil}}$ of the sensor due to humidity vaporization. The resistance of copper wire changes linearly with the temperature. In combination with the skin effect, the current density changes. This leads to small changes in the inductance $L_{\mathrm{Coil}}$ of the sensing coil. The thermal expansion of the former affects the length *a* and width *b* of the coil and thus the effective cross-sectional area A=a·b. For a solenoid coil, the inductance can be described as $L∼\mu_{0}\mu_{r}N^{2}A/l$. The thermal expansion is considered linear, described by $a(1+\alpha_{1}\Delta \vartheta_{\mathrm{Coil}})$ for the length and $b(1+\alpha_{2}\Delta \vartheta_{\mathrm{Coil}})$ for the width. The change in height of our sensing coil is negligible and the thermal expansion is dominant compared to the electrical conductivity change; thus, ΔL∼ΔA. The composite temperature compensation model of the sensing coil is expressed as

(4) $$\begin{array}{c} L_{\mathrm{Coil}}=L_{0}\left(1+\alpha \Delta \vartheta_{\mathrm{Coil}}+\beta \Delta \vartheta_{\mathrm{Coil}}^{2}\right), \end{array}$$

where $L_{0}$ is the inductance of the sensing coil in air (in the absence of the target) at room temperature, and α and β are the temperature coefficients. $\vartheta_{\mathrm{Coil}}$ is the temperature of the sensing coil. The temperature model assumes a uniform temperature distribution across the coil former in a stationary state, with negligible local temperature gradients. This was considered in the experiments. Furthermore, with minor vaporization effects, the changes in parasitic capacitance $C_{\mathrm{Coil}}$ are negligible. Hysteresis effects, although present, are not taken into account in the model but are discussed in the summary.

### 4.2. Temperature Variations—Experiments with the Gypsum and Fired Clay-Based Formers

For the analysis, we use the lab setup shown in Figure 16. The sensing coil is positioned within a thermal box, ensuring minimal interference from conductive materials within the sensing range. This setup allows for controlled heating of the sensing coil from room temperature up to 130 °C. In this temperature range, we also check the validity of the temperature compensation. The temperature of the sensing coil is measured with thermocouples. For the temperature characterization, we approached several temperatures, where the sensing coil reaches a steady-state temperature, up to 130 °C, at frequencies of 500 Hz and 15 kHz. All the heating experiments for the sensing coil were conducted sequentially.

**Figure 17:**
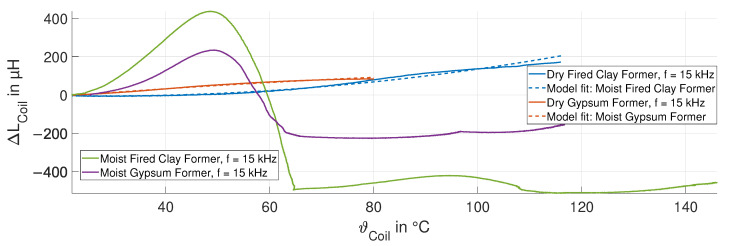
Measured change in inductance of the sensing coil $\Delta L_{\mathrm{Coil}}$ of the moist and dry fired clay and gypsum formers at 15 kHz, and the fits of the temperature compensation model.

The change in the inductance $\Delta L_{\mathrm{Coil}}$ due to the temperature variations for both the dry and moist coil formers for one experiment are shown in Figure 17 at a frequency of 15 kHz.

The change in the inductance $\Delta L_{\mathrm{Coil}}$ for the coil on the dry fired clay (blue curve) and dry gypsum (red curve) formers increases with the temperature. This behavior was confirmed by a field simulation in COMSOL Multiphysics^®^. The change in the inductance $\Delta L_{\mathrm{Coil}}$ can be represented by the temperature compensation model in (4), as the fits (dashed curves) indicate in Figure 17. However, with repeated measurements, i.e., repeated heatup and cooldown cycles, the behavior of the inductance changed slightly. This is due to different humidity contents in the former and effects of vaporization. When heated, humidity evaporates from the former, changing the impedance of the coil $Z_{\mathrm{Coil}}$. The effect of humidity vaporization on the inductance during heating can be significant if the fired clay and gypsum formers were stored in the lab prior to the experiments, where they were exposed to the environmental humidity and absorbed humidity. The purple and green curves in Figure 17 show the initial heat cycling experiments for the clay and gypsum formers after long exposure to humidity.

**Figure 18:**
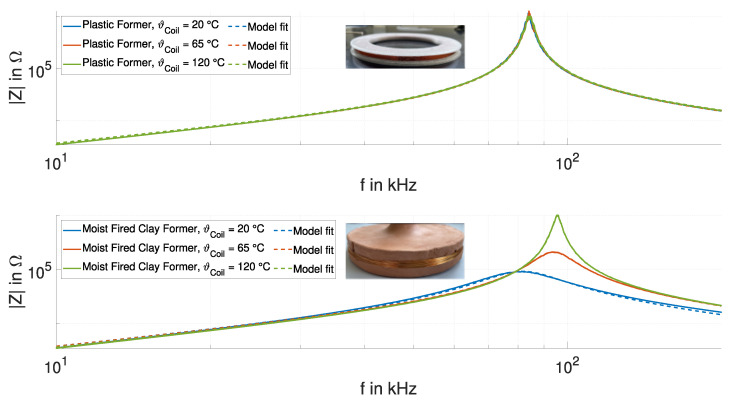
Impedance characteristics of the coils on the plastic former (**upper subplot**) and the fired clay former (**lower subplot**) during heating, and the model fits based on a parallel RLC circuit.

Using a coil prototype on a nonhygroscopic plastic former, we demonstrate the insensitivity of the inductance $L_{\mathrm{Coil}}$ to humidity vaporization. The plastic prototype has the same winding cross-section as the fired clay former. We compare the impedance characteristics to the coil on the moist clay former. The impedance spectroscopy measurements (solid curves), a model fit based on a parallel RLC circuit (dashed curves), and a photo of the plastic coil former as the inset are shown in the upper subplot in Figure 18. The measured impedance trends are on top of each other, indicating minimal temperature-induced variations in the resonance behavior. The resonance behavior can be described with the parallel RLC circuit. The fits match the measured impedance trends and all the parameters hardly change.

The lower subplot in Figure 18 shows the impedance spectroscopy measurements and model fits for the moist fired clay former. The resonance frequency and the quality factor significantly change with increasing temperature. Variations in the temperature and humidity change the dielectric permittivity and the electrical conductivity of the clay former [28], resulting in changes in the parasitic capacitance $C_{\mathrm{Coil}}$ [29]. Losses in the former changes [29], leading to changes in the quality factor. For these experiments, the plastic and clay formers were exposed to the same humidity for the same period of time.

The impedance trends for the coil on the moist fired clay former converge below the resonance frequency, but there remains an influence at lower frequencies. The minor deviation between the measured resonance behavior and the model fit increases with the humidity content. Despite the deviation, the model is suitable for describing the resonance behavior and the change in the parameters of the RLC circuit with the temperature, listed in Table 2. From the dry to the moist formers, the parasitic capacitance changes by 30% and the resistance by 90%, while the inductance changes by less than 5%. The change in inductance is comparable to the results of the experiment in Figure 17.

**Figure 19:**
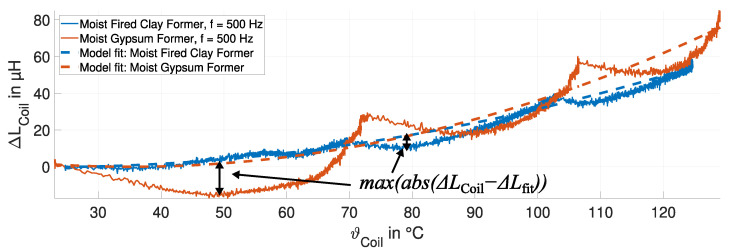
Measured change in inductance of the sensing coil $\Delta L_{\mathrm{Coil}}$ (solid curves) and model fit (dashed curves) for the fired clay (blue) and gypsum (red) formers at 500 Hz.

One approach to reduce the impact of humidity variations on the inductance $L_{\mathrm{Coil}}$ of the sensing coil is to reduce the measurement frequency, e.g., to 500 Hz. The change in inductance of the sensing coil $\Delta L_{\mathrm{Coil}}$ on the moist fired clay and the moist gypsum former is shown in Figure 19. The fits of the temperature compensation model (4) are also plotted.

Both trends show an increase in the change in inductance $\Delta L_{\mathrm{Coil}}$ with increasing temperature. The effect of humidity vaporization on the inductance $L_{\mathrm{Coil}}$ is less than half of that at 15 kHz and is dominated by thermal expansion. This is confirmed by the field simulation study. The change in the inductance $\Delta L_{\mathrm{Coil}}$ is more pronounced for gypsum due to the higher coefficient of the thermal expansion coefficient of gypsum (25 × 10^−6^ 1/K) compared to fired clay (7.5 × 10^−6^ 1/K). The initial decrease in the change in the inductance $\Delta L_{\mathrm{Coil}}$ is attributed to vaporization. The temperature compensation model can describe the behavior of the inductance $L_{\mathrm{Coil}}$. However, the temperature compensation model also has limitations, particularly during the heating phase between the stationary temperatures, i.e., marked by the two errors. This is due to more pronounced humidity vaporization from the coil former at the beginning of the temperature increase.

### 4.3. Coil: Temperature-Induced Inductance Variations—Determination of the Position Error

We demonstrate the effectiveness of the proposed temperature model in Equation (4) based on the position error $e_{\mathrm{pos}.}$. The performance of the temperature model is evaluated for high temperature variations, extending from room temperature to 120 °C over the entire displacement range from 20 mm to 100 mm at a frequency of 500 Hz.

First, we calculate the change in the inductance $\Delta L=L\left(\vartheta_{\mathrm{Coil}}\right)-L_{0,x=20\mathrm{mm}}$, where $L(\vartheta_{\mathrm{Coil}})$ is calculated via Equation (2). To translate the change in the inductance ΔL into a quantifiable position $x_{\mathrm{Coil}}=\Delta L/S$, we use the sensitivity *S*. With this, we calculate the position error $e_{\mathrm{pos}.}=x_{\mathrm{Coil}}-x_{\mathrm{Ref}.}$, where $x_{\mathrm{Ref}.}$ is the reference position of the stepper motor.

**Figure 20:**
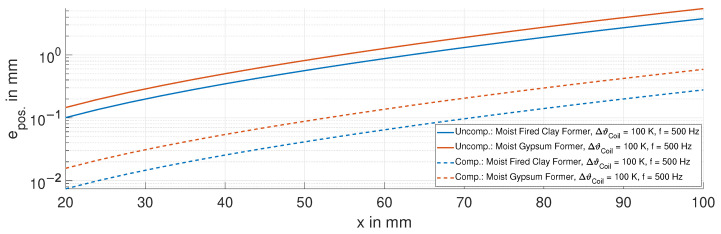
Position error $e_{\mathrm{pos}.}$ for the uncompensated and compensated cases, for a temperature variation in the sensing coil of 100 K at 500 Hz.

Figure 20 shows the position error $e_{\mathrm{pos}.}$ for both former materials, comparing the uncompensated (solid curves) and compensated (dashed curves) cases. The temperature model reduces the position error to just 10% of that in the uncompensated case.

## 5. Calibration Approach and Determination of the Position Error

In this section, we present a calibration approach based on the sensor model in Equation (2) and the field simulation. We show the potential of the coupling coefficient k(x) for determining the position *x*. Given that the fraction term $f_{T}$ for copper at 15 kHz is close to that of a superconductor (nearly 1), we use the coupling coefficient k(x) from the field simulation for calibration. Therefore, we apply the following offset-gain calibration, involving a shift and scaling of the coupling coefficient k(x)

(5) $$\begin{array}{c} \hat{k}\left(x,p\right)=p_{3}\;\cdot \;k\left(\frac{x-p_{1}}{p_{2}}\right)+p_{4}. \end{array}$$

**Figure 21:**
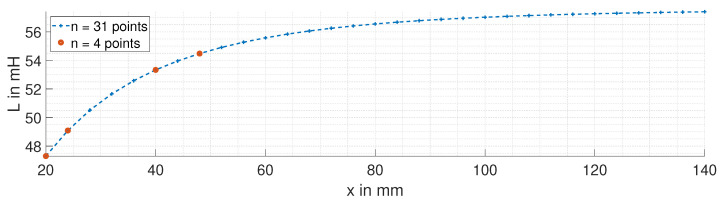
Chosen measurement points, n=31 and n=4, for the calibration process.

The parameters *p* are estimated by solving the following problem

(6) $$\begin{array}{c} \hat{p}=\underset{p}{\mathrm{argmin}}\sum_{i=1}^{n}{\left(L_{\mathrm{meas},i}-L_{\mathrm{Coil}}\;\cdot \;\left(1-{\hat{k}}_{i}{\left(x,p\right)}^{2}\right)\right)}^{2}, \end{array}$$

using a stochastic optimizer. The calibration of the system was performed with two distinct sets of measurement points as shown in Figure 21. The first set consists of n=31 measurement points, which covers a wide operating range of the sensor. This provides a fidelity in the calibration process but at the cost of a longer calibration time. The second set uses a significantly reduced number of measurement points (n=4), which shortens the calibration process. The position error $e_{\mathrm{pos}.}$ is used to evaluate the two calibration strategies. The second set is exceptional because we selected the points to be in a region of the ECDS with higher sensitivity. Including the start and end points is usually recommended.

With the calibration complete, we evaluate the impact of the temperature variations in the target $\Delta \vartheta_{T}$ and the sensing coil $\Delta \vartheta_{\mathrm{Coil}}$ on the position *x*. This evaluation is based on the sensor model in Equation (2), where we assume $f_{T}=1$, a valid assumption when using the copper target. This leads to the following equations:

- Target: $L_{\mathrm{ECDS}}\left(\vartheta_{T}\right)=L_{\mathrm{Coil}}\left(\vartheta_{\mathrm{Coil}}=\mathrm{const}.\right)\;\cdot \;\left(1-\tilde{k}\left(x\right)\right)$, where the temperature of the target varies ($\Delta \vartheta_{T}$) and the sensing coil is at a constant temperature ($\vartheta_{\mathrm{Coil}}=\mathrm{const}.$);
- Sensing coil: $L_{\mathrm{ECDS}}\left(\vartheta_{T}=\mathrm{const}.\right)=L_{\mathrm{Coil}}\left(\vartheta_{\mathrm{Coil}}\right)\;\cdot \;\left(1-\tilde{k}\left(x\right)\right)$, where the temperature of the sensing coil varies ($\Delta \vartheta_{\mathrm{Coil}}$) and the target is at a constant temperature ($\vartheta_{T}=\mathrm{const}.$).

The measurement data for the inductance *L* and the inductance of the sensing coil $L_{\mathrm{Coil}}$ are based on the experiments in Section 3 and Section 4. We use the setup with the fired clay former and the copper target. Those two equations above are transformed to $\tilde{k}\left(x\right)$. We determine the estimated position $\tilde{x}$ by performing a numerical interpolation based on the calculated $\tilde{k}\left(x\right)$ and known $\hat{k}\left(x\right)$ values. The position error is then quantified by $e_{\mathrm{pos}.}=\tilde{x}-x_{\mathrm{Ref}.}$.

**Figure 22:**
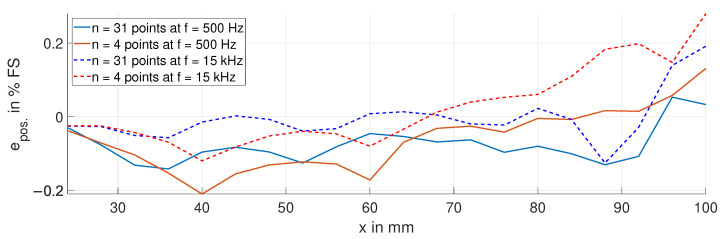
Position error $e_{\mathrm{pos}.}$ due to a temperature variation in the target, with $\Delta \vartheta_{T}$ = 110 K, at 500 Hz and 15 kHz for both sets of measurement points.

Figure 22 shows the position error $e_{\mathrm{pos}.}$ for the calibration with both sets of measurement points at frequencies of 500 Hz and 15 kHz. This is for a temperature variation in the target of 110 K. The position error ranges from −0.12% FS to 0.2% FS, and the TS ranges from −11 ppm FS/K to 18 ppm FS/K. The reduced number of measurement points does not result in a considerably increased position error.

**Figure 23:**
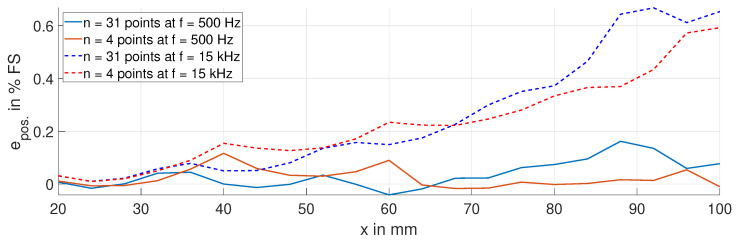
Position error $e_{\mathrm{pos}.}$ due to a temperature variation in the sensing coil, with $\Delta \vartheta_{\mathrm{Coil}}$ = 100 K, at 500 Hz and 15 kHz. Both sets of measurement points, including the temperature compensation.

Figure 23 shows the corresponding position errors $e_{\mathrm{pos}.}$ resulting from temperature variations in the sensing coil of 100 K. This analysis includes compensation via the temperature model in Equation (4). At 15 kHz, the position errors $e_{\mathrm{pos}.}$ are larger than at 500 Hz, despite the compensation measures. This is due to the vaporization of humidity during the heating of the fired clay former, which alters the parasitic capacitance $C_{\mathrm{Coil}}$. At 500 Hz, this effect is less pronounced on the inductance *L*, resulting in a position error $e_{\mathrm{pos}.}$ below 0.2% FS and the TS below 22 ppm FS/K. Moreover, there is only a minor difference in the position error between the two sets of measurement points.

The achieved TS is comparable to, or exceeds, that of both the commercially available ECDSs (Table 1) and published academic research studies on ECDSs (Table A1).

## 6. Summary: Considerations for Harsh Environments

To reduce the influence of temperature variations in the target on the inductance *L*, there are two options with respect to $f_{T}$ in Equation (2). First, with a highly conductive material of the target, such as copper, the time constant $\tau_{T}$ is larger. Second, an increase in the measurement frequency gets $f_{T}$ closer to 1 (properties of a superconductor). When increasing the measurement frequency, it is necessary to balance the greater insensitivity to changes in the material properties of the target against the impact of the temperature variation in the sensing coil on the inductance *L*, especially when using hygroscopic materials. Regardless of whether the frequency or $\tau_{T}$ is increased, which is limited by the material properties, there will always remain a sensitivity, although minimal, to the target.

The material for the coil former should withstand high temperatures and have minimal thermal expansion, low humidity absorption, and minimal eddy current effects. Fired clay is suitable for high temperature applications due to its temperature resistance and ease of manufacture. However, its porosity leads to significant humidity absorption and vaporization during heating, changing the parasitic capacitance $C_{\mathrm{Coil}}$, the resistance $R_{\mathrm{Coil}}$, and the inductance $L_{\mathrm{Coil}}$ and hence the inductance *L*.

When using a fired clay-based former, we suggest coating it with a porcelain glaze to make it impermeable to liquids, thereby decreasing its humidity absorption. Not the entire coil former contributes equally to the parasitic effects. Therefore, a nonhygroscopic spacer in which the windings are wound could be considered. A second option is to use a lower measurement frequency, which reduces parasitic effects. However, this approach involves a trade-off for ECDS applications, as the influence of changes in the target material properties on the inductance *L* increases at lower measurement frequencies [9].

We recommend using a technical ceramic, such as aluminum oxide (${\mathrm{Al}}_{2}O_{3}$) or silicon carbide (SiC), or a glass ceramic, like Macor^®^ or Mica^®^ for the intended application. However, the use of such materials is coupled with an extensive manufacturing process.

To compare different ECDS prototypes, it is recommended to use the relative change in inductance $\Delta L/L_{x_{\mathrm{Ref}.}}$, where $L_{x_{\mathrm{Ref}.}}$ is the inductance at a reference position. Given the relative change in inductance, we found that reducing the number of turns results in minimal degradation over the displacement range. In addition, using a single-layer coil, which reduces the parasitic capacitance $C_{\mathrm{Coil}}$, would further increase the self-resonance frequency (SRF). Thus, a higher measurement frequency can still be one order of magnitude below the SRF but would greatly increase the insensitivity of the inductance *L* to changes in the material properties of the target.

## 7. Conclusions

In this study, we have analyzed the properties and suitability of a single-coil eddy current displacement sensor (ECDS) design for use in harsh environments. This analysis was conducted using a sensor model based on a tailored equivalent circuit model (ECM) incorporating the effects of temperature, humidity, and displacement. Our findings highlight the substantial impact of high temperature variations and humidity on the inductance *L* and, consequently, the position error $e_{\mathrm{pos}.}$. We provide guidance for users to tailor the sensor design, regarding the relevant material selection for the sensing coil and the target, to their specific environmental conditions. A temperature compensation model is proposed to reduce the effects of temperature variations on the inductance of the sensing coil $L_{\mathrm{Coil}}$. Additionally, we show that a calibration based on the sensor model in Equation (2) is feasible, even with a reduced number of measurement points. The coupling coefficient k(x) for determining the position *x* can even be derived from simulation data. Specifically, for the sensing coil on a fired clay former, the position error $e_{\mathrm{pos}.}$ remains below 0.2% FS for a temperature variation in the sensing coil of 100 K at 500 Hz. Similarly, for a temperature variation in the target of 110 K, the position error $e_{\mathrm{pos}.}$ is maintained under 0.2% FS. The proposed design surpasses commercially available ECDS designs in similar displacement and temperature ranges. It is surpassed by other academic research study designs on ECDSs with comparable temperature variations. However, the displacement range of these studies is smaller by a factor of 100. Future studies will address the coupled effects and interactions of the sensing coil and the target, as well as practical implications, such as long-term stability, and improvements in the former material. This study serves as a basic guide for initial system optimization of ECDSs in various applications.

## Figures and Tables

**Table 1:** Summary of key findings from comparative commercial ECDSs for harsh environments.

Manufacturer	Range *x*	Diameter	Temperature	Temperature Stability
Micro Epsilon EU80	80 mm	140.3 mm	−40 to 200 °C	<150 ppm FS/K
Kaman KDM-8206	60 mm	152.4 mm	0 to 55 °C	Standard: 1000 ppm FS/K, Comp.: 200 ppm FS/K
ATO ECDS-5060	50 mm	60 mm	−30 to 150 °C	Standard: 1000 ppm FS/K, Comp.: 200 ppm FS/K
This Work	100 mm	150 mm	20 to 130 °C	Comp.: Coil: <22 ppm FS/K, Target: −11 to 18 ppm FS/K

**Table 2:** Fitted RLC circuit parameters for the measured resonance behavior of the coil.

$\vartheta_{\mathrm{Coil}}$	$L_{p}$	$C_{p}$	$R_{p}$
20 °C	61 mH	62.5 pF	92 kΩ
65 °C	60.4 mH	47.46 pF	212 kΩ
120 °C	58.4 mH	47.53 pF	984 kΩ

## Data Availability

Data are contained within the article.

## Appendix A. Eddy Current Displacement Sensors in Harsh Environments

### Comparison of Academic Research Studies

We present a comparative analysis of four academic research studies on ECDSs, focusing on applications involving large displacements and high temperature variations. Each study is analyzed based on its sensor design, material selection, and compensation methods. We evaluate their performance based on the temperature stability (TS). The key findings from these studies are listed in Table A1 and discussed below.

**Table A1:** Summary of key findings from comparative academic research studies analyzing ECDSs in harsh environments.

Ref.	Range *x*	Diameter	Temp.	Temp. Stability	Comment
(a), [8]	2 mm	$d_{i}$ = 2 mm $d_{o}$ = 4 mm	ϑ ∼ 18 °C ± 3 °C Δϑ ∼ 6 K	1.5 ppm FS/K 3 nm/K	Discussion of coil properties; incl. ref. coil for temp. compensation; detection in sub nm range
(b), [14]	40 mm	d = 11 mm	ϑ ∼ 30 °C Δϑ ∼ 12 K	3000 ppm FS/K 120 μm/K	Use of one excitation coil and two sensing coils
(c), [18]	2 mm	not specified	ϑ ∼ 300 °C Δϑ ∼ 320 K	1.3 mm: 170 ppm FS/K	Compensation probe and a compensation plate to feedback exponential hysteresis temp. drift error
(d), [30]	±2.5 mm	not specified	ϑ = −40 to 50 °C Δϑ ∼ 90 K	±13.7 ppm FS/K ±69 nm/K	Use of a differential sensor probe to reduce the influence of temp. drift

For small displacements: Reference (a) in Table A1 [8] focuses on distinguishing sub nm displacement variations and uses a reference coil to handle temperature drifts, achieving a high TS. However, the lift-off of the sensing coil is not specified, which is a crucial factor because multiple studies show that the TS varies with the lift-off [19,20,21].

For large displacements: The authors of [14,15] use a setup with multiple coils to enhance the sensitivity of the ECDS, which is just one approach. For example, reference (b) in Table A1 [14] uses a constellation of multiple coils for displacements that far exceed the coil diameter (x∼4·d). This improves the linearity and thermal drift coefficient (3000 ppm/K). However, the large displacement range and the higher temperature variation, compared to reference (a), significantly degrade the TS.

For high temperature and temperature variations: Reference (c) in Table A1 [18] proposes a mechanical design solution. They use a compensation probe and a compensation plate to reduce exponential hysteresis drift errors, achieving a TS of 170 ppm FS/K at a lift-off of x=1.3 mm [18]. However, the study lacks details on sensor dimensions, and it does not address how to correct exponential hysteresis temperature drift errors when the temperatures of the working probe and compensation probe are out of synchronization [18].

For temperature variations between 12 K and 320 K, (between reference (b) and (c)), reference (d), [30], develops a low-temperature-drift differential-digital demodulation sensor. The study also lacks details on sensor dimensions. Nevertheless, the TS shows an improvement by a factor of 10 compared to reference (c) due to the temperature variation being about one-third smaller.
